# Efficacy of Transcranial Magnetic Stimulation for Reducing Suicidal Ideation in Depression: A Meta-Analysis

**DOI:** 10.3389/fpsyt.2021.764183

**Published:** 2022-01-18

**Authors:** Yanan Cui, Haijian Fang, Cui Bao, Wanyue Geng, Fengqiong Yu, Xiaoming Li

**Affiliations:** ^1^School of Mental Health and Psychological Sciences, Anhui Medical University, Hefei, China; ^2^School of the First College for Clinical Medicine, Anhui Medical University, Hefei, China; ^3^Anhui Province Key Laboratory of Cognition and Neuropsychiatric Disorders, Department of Mental Health and Psychological Science, The Second Affiliated Hospital of Anhui Medical University, Anhui Medical University, Hefei, China

**Keywords:** transcranial magnetic stimulation, suicidal ideation, meta-analysis, depression, TMS

## Abstract

**Objectives:**

This study aimed to systematically review the efficacy of transcranial magnetic stimulation treatment in reducing suicidal ideation in depression.

**Methods:**

PubMed, Web of Science, CBMdisc, WanFang, Chongqing VIP, and CNKI databases were electronically searched for randomized controlled trials of transcranial magnetic stimulation (TMS) intervention in the management of suicidal ideation from inception to February 24, 2021. Two reviewers independently screened studies, extracted data, and assessed the quality of included studies. Meta-analysis was then performed using STATA 15.1 software.

**Results:**

A total of eight articles involving 566 patients were included. The meta-analysis results showed that the suicidal ideation scores of the group who received TMS treatment were significantly lower [standardized mean difference (SMD) = −0.415, 95% confidence interval (CI): −0.741 to −0.090, *P* = 0.012] than those of the control group. Subgroup analysis showed that age, TMS pattern, frequency of intervention, and stimulation threshold altered the TMS efficacy.

**Conclusions:**

Evidence showed that TMS achieved superior results in reducing suicidal ideation. Because of the limited quality and quantity of the included studies, more high-quality studies are required to verify the conclusions.

**Systematic Review Registration:**

https://inplasy.com/, identifier: INPLASY202180065.

## Introduction

Major depressive disorder (MDD) is a serious, worldwide mental issue, influencing millions of individuals (1). More than 50% of Chinese patients with MDD have suicidal ideation (2). Suicide is not only a major health problem but also a social problem (3). According to global data released by the World Health Organization in 2012, more than 800,000 people die by suicide every year, accounting for 1.4% of the world's death toll and making it the 15th leading cause of death (4, 5). The lifetime prevalence of suicide ideation is approximately 9.2% on a global scale (6). Suicidal ideation is defined as thinking about, considering, or planning suicide (6). In a review of the ECT intervention literature, Fink et al. found that ECT was effective for individuals with major depression and suicide, and for follow-up (7). Cipriani et al. found that the Li intervention group reduced suicide deaths by at least 60% compared to the control group (8). Accessible psychological and pharmacological interventions have meant that advancements have been made in reducing suicide (9); however, these are not without side effects, which influences their effectiveness and may further negatively affect those already at high risk of suicide (10). Hence inventive treatment procedures to prevent suicide, for use alongside existing treatments, are fundamentally required.

There is a growing interest in the use of non-invasive brain incitement techniques to decrease suicidal intent and behavior. Transcranial magnetic stimulation (TMS) is a non-invasive magnetic stimulation technology in which a pulsed magnetic field acts on the central nervous system (mainly the brain) to change the membrane potential of cortical nerve cells to produce induced current, which affects brain metabolism and nerve electrical activity, and causes a series of physiological and biochemical reactions (11). A form of TMS, intermittent TBS (iTBS), actuates a long-term potentiation (LTP)-like impact by expanding the postsynaptic concentration of calcium particles (12). Both TBS and traditional patterned TMS can induce plastic changes in a parameter-dependent manner (e.g., inhibit or excite as a function of frequency). The physiological and therapeutic antidepressant magnitude of the effect seem to be similar. Physiological studies have shown that the duration of the physiological modulation on the motor cortex may be longer for TBS than patterned TMS. It is not clear that this translates to clinical efficacy though (in fact, the antidepressant benefits may be shorter) (13–16). TBS has been shown to be safe and well-tolerated, and to have antidepressant properties (17). A recent study (18) suggests that TBS is efficacious in suicide.

Other studies have discussed that rTMS can affect the emotional and cognitive state of patients (19), and that patients with suicidal ideation and behavior often have damage to areas of the brain that are involved in cognitive and emotional control functions (20, 21), and that the targets of TMS happen to be implicated in these brain areas (22), which lends support to the idea that TMS would be expected to be an effective treatment for suicide and its potential use as a treatment for suicidal ideation.

Some studies (23) have not demonstrated a significant difference in the reduction of suicide scores between active TMS stimulation and sham stimulation. By comparing the effects of epilepsy treatment and non-invasive brain stimulation on suicide, Chen et al. supported the effect of ECT on acute suicidal ideation, but they could not suggest the same for MST, rTMS, or tDCS (24). Bozzay et al. review (25) supports the ongoing use of TMS as a new medium to reduce suicide risk; Serafini et al. analyzed the relationship between rTMS interventions and suicidal behavior, one of multiple suicidal dimensions (e.g., suicidal ideation, intensity of suicidal thoughts, suicidal behavior, and suicidal intent), in a systematic study (26). A Meta-analysis (27) concluded that the efficacy and efficiency of high frequency was higher than that of the sham stimulation group; Brunoni et al. (28) included nearly 100 studies and showed that low frequency stimulation was the most effective, while high frequency stimulation was the least effective, bilateral stimulation was intermediate, and bilateral stimulation and low frequency stimulation were the most acceptable of the stimulation modes; Dell'Osso et al. (29) showed that high-frequency stimulation and low-frequency stimulation were similarly effective. The treatment is also effective in special populations, especially adolescents, but the follow-up and delayed effects of the treatment are also of concern in adolescents who are not fully neurologically mature. However, few meta-analyses support the use of TMS for suicidal ideation (one of multiple suicidal dimensions) interventions or provide insight into how best to develop and utilize such interventions. To fill this knowledge gap, we conducted a meta-analysis on the efficacy of TMS in the treatment of suicidal ideation, with subgroup analyses of TMS patterns, age, stimulation frequency and intensity.

## Methods

The study has been registered on INPLASY website. The registration number of this meta-analyses protocol is INPLASY202180065.

### Search Strategy

Six electronic databases were searched for relevant studies: PubMed, Web of Science, WanFang, Chinese National Knowledge Infrastructure, Chongqing VIP, and CBMdisc, from their establishment to February 24, 2021, with no restrictions on the publication year. The word “suicide” was combined with “transcranial magnetic stimulation” and the search strategy of combining subject words with free words was adopted (Supplementary Materials). Studies were assessed by the inclusion and exclusion criteria below and sorted first by examination of title, then abstract, then the full text. The final search of each database was performed independently and separately by two reviewers.

### Inclusion Criteria

The selected studies were those that met the following eligibility criteria: (1) randomized controlled trials published in English or Chinese; (2) the age of participants ranged from 13- to 80-years-old; (3) the study group was treated with TMS or a physical intervention with a definite treatment plan, including a different sequence and frequency of neurophysical stimulation; (4) the control group had no restrictions in the treatment they received (except other physical treatments such as ECT,TDCS, etc.), including conventional treatment, placebo treatment, and waiting for treatment; and (5) the evaluation results were of suicidal ideation and suicidal behavior.

### Data Extraction

Information was extracted independently by two reviewers in a standardized manner. Any disagreements were discussed with another reviewer, to reach consensus. Engauge Digitizer 12.1 was used to obtain more information [Only one study (30) in the included studies did not give the data we needed, but we extracted them from the figure by means of the tool]. The following data were extracted for each study: first author's name, year of publication, location, sample size, psychometric instruments and mean and standard deviation of suicide ideation score. We also extracted information on sample size, age, type of TMS, intervention frequency, and intensity threshold, in order to estimate TMS efficacy for suicidal ideation by subgroups.

### Quality Assessment

Cochrane risk of bias assessment (31) was used to evaluate the study quality according to the following criteria: random sequence generation, allocation concealment, blinding, incomplete outcome data, selective reporting, and other sources of bias. Each area was ranked for high, low, or unknown bias risk. We also calculated the Jadad score for each of the included studies (32). In calculating the Jadad score, each study was evaluated according to the quality of randomization, blinding procedures, and description of withdrawals and dropouts. Jadad scores ranged from 0 to 5, with trials scoring 3 or greater considered good quality trials.

### Statistical Analysis

WPS Office 3.0.2 software was used to organize the incorporated literature and data, and statistical analysis was completed using STATA version 15.1 software. The Q test was used to explore the variation between studies. The *I*^2^ statistic reflected the proportion of heterogeneity in the total variation of effect. If the heterogeneity test results were *P* > 0.1 and *I*^2^ < 50%, the homogeneity of the included studies was considered to be good, and a fixed effects model was used; otherwise, a random effects model was used. Publication bias was assessed by a funnel plot and Egger's test. Subgroup analysis was conducted to explore the potential heterogeneity between studies and the efficacy of TMS intervention for suicidal ideation according to different characteristics.

## Results

### Characteristics of Eligible Studies

**Figure 1:**
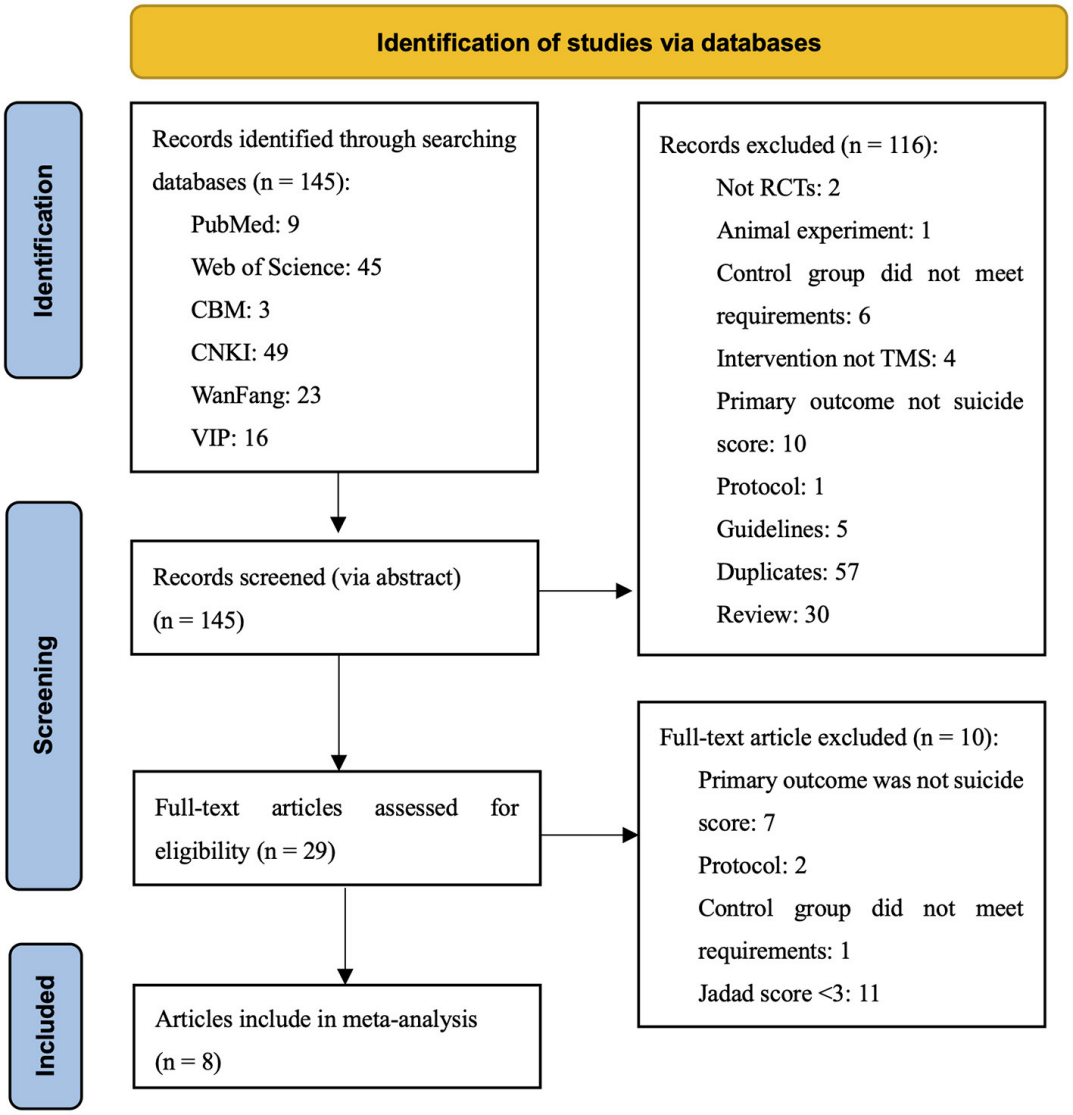
PRISMA flow diagram of the systematic review phases.

Through searching, 145 potentially related articles were found. After the title and abstract were screened, a remaining 29 documents were screened for full text. Finally, eight articles met the inclusion criteria for meta-analysis. Two studies within one article met the inclusion criteria, making a total of nine studies for meta-analysis. The document screening process and results are shown in Figure 1.

The baseline information from the included studies is presented in Table 1. Nine sham-controlled clinical trials, including a total of 566 patients, 7 rTMS-controlled clinical trial including a total of 490 patients, 2 iTBS-controlled clinical trial including a total of 76 patients were included in the present systematic review. Clinical samples included predominantly patients with suicidal ideation and one of the following psychiatric diagnoses: MDD, treatment resistant depression (TRD). Subjects in all studies were taking antidepressants during treatment, except for Stefanie et al. Chris et al. (Only habitual benzodiazepine agents were allowed), two studies went through a drug washout period.The control group in all studies was given a sham stimulation treatment, i.e., the same coil also emits a tapping sound on the surface of the patient's scalp, but without pulses. The Jadad score for all the included studies was ≥3. The results of the bias risk assessment are shown in Table 2.

**Table 1:** Characteristics of eligible studies included in the meta-analysis.

**References**	**Participants**	**Sample size (T/C)**	**Age**	**TMS pattern**	**Target**	**Frequency**	**Intensity (% MT)**	**Sessions/day**	**Number of Pulses per session**	**Total number of TMS sessions**	**Control group**	**Psychometric instruments**
Mark et al. (30)	Depressed Adults hospitalized for suicidality with PTSD and/or mild TBI	20/21	<50	rTMS	L DLPFC	high	120%	3 sessions/day	6,000	9 sessions	Sham stimulation and TAU	SSI
Stefanie et al. (33)	MDD unipola, antidepressant-free patients	14/18	<50	iTBS	L DLPFC	high	110%	5 sessions/day	1,620	20 sessions	Sham stimulation	BSI
Chris et al. (34)	TRD, antidepressant-free patients	18/26	<50	iTBS	L DLPFC	high	110%	5 sessions/day	1,620	20 sessions	Sham stimulation and TAU	SSI
Jerome et al. (23)	inpatients with TRD, taking antidepressants.	73/77	≥50	rTMS	L DLPFC	high	120%	2–6 sessions/day	4,000	20–30 sessions	Sham stimulation and TAU	BSI
Qi (35)	MDD, taking antidepressants.	30/30	<50	rTMS	L DLPFC	high	100%	1 sessions/day	1,500	10 sessions	Sham stimulation and TAU	SSI
Qi (35)	MDD, taking antidepressants.	32/30	<50	rTMS	R DLPFC	Low	100%	1 sessions/day	1,500	10 sessions	Sham stimulation and TAU	SSI
Junbo (36)	adolescents with depression, taking antidepressants	16/16	<50	rTMS	R DLPFC	Low	80%	1 sessions/day	1,000	10 sessions	Sham stimulation	BSI
Lilei et al. (37)	elderly patients with depression and suicidal ideation, taking antidepressants	48/55	≥50	rTMS	L DLPFC	high	100%	1 sessions/day	800	20 sessions	Sham stimulation and TAU	SIOSS
Fen et al. (38)	MDD, taking antidepressants	21/21	<50	rTMS	L DLPFC	high	100%	1 sessions/day	6,000	7 sessions	Sham stimulation	BSI

*rTMS, repetitive transcranial magnetic stimulation; iTBS, intermittent Theta Burst Stimulation; SIOSS, self-rating idea of suicide scale; BSI/SSI, Beck Scale for Suicide Ideation; DLPFC, Dorsolateral prefrontal cortex; L, Left; R, Righ; MDD, major depressive disorder; TRD, Treatment-Resistant Depression; TAU, Treatment As Usual*.

**Table 2:** Quality assessment of included studies.

**References**	**Random sequence generation**	**Blinding**	**Allocation concealment**	**Incomplete outcome data**	**Selective reporting**	**Other sources of bias**
Fen et al. (38)	Low risk	Low risk	Unclear	Low risk	Low risk	Low risk
Jerome et al. (23)	Low risk	Low risk	Low risk	Low risk	Low risk	Low risk
Lilei et al. (37)	High risk	Low risk	Unclear	Low risk	Low risk	Low risk
Junbo (36)	Low risk	Low risk	Unclear	Low risk	Low risk	Low risk
Qi (35)	Low risk	Unclear	Unclear	Low risk	Low risk	Low risk
Chris et al. (34)	High risk	Low risk	High risk	Low risk	Low risk	Low risk
Stefanie et al. (33)	High risk	Low risk	High risk	Low risk	Low risk	Low risk
Mark et al. (30)	High risk	Low risk	Low risk	Low risk	Low risk	Low risk

### Overall Efficacy of TMS

#### Suicidal Ideation

**Figure 2:**
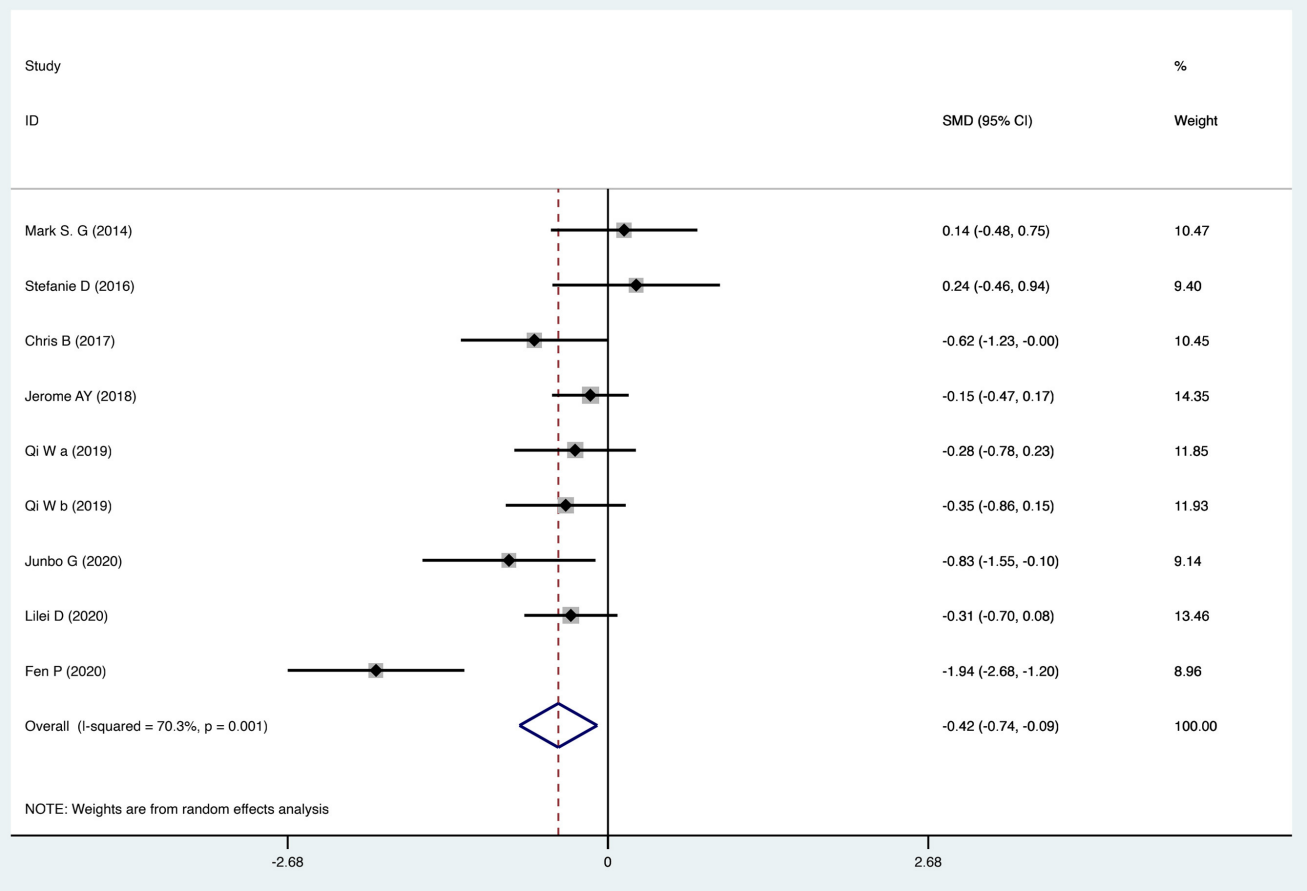
Forest plot of transcranial magnetic stimulation intervention for suicidal ideation.

**Figure 3:**
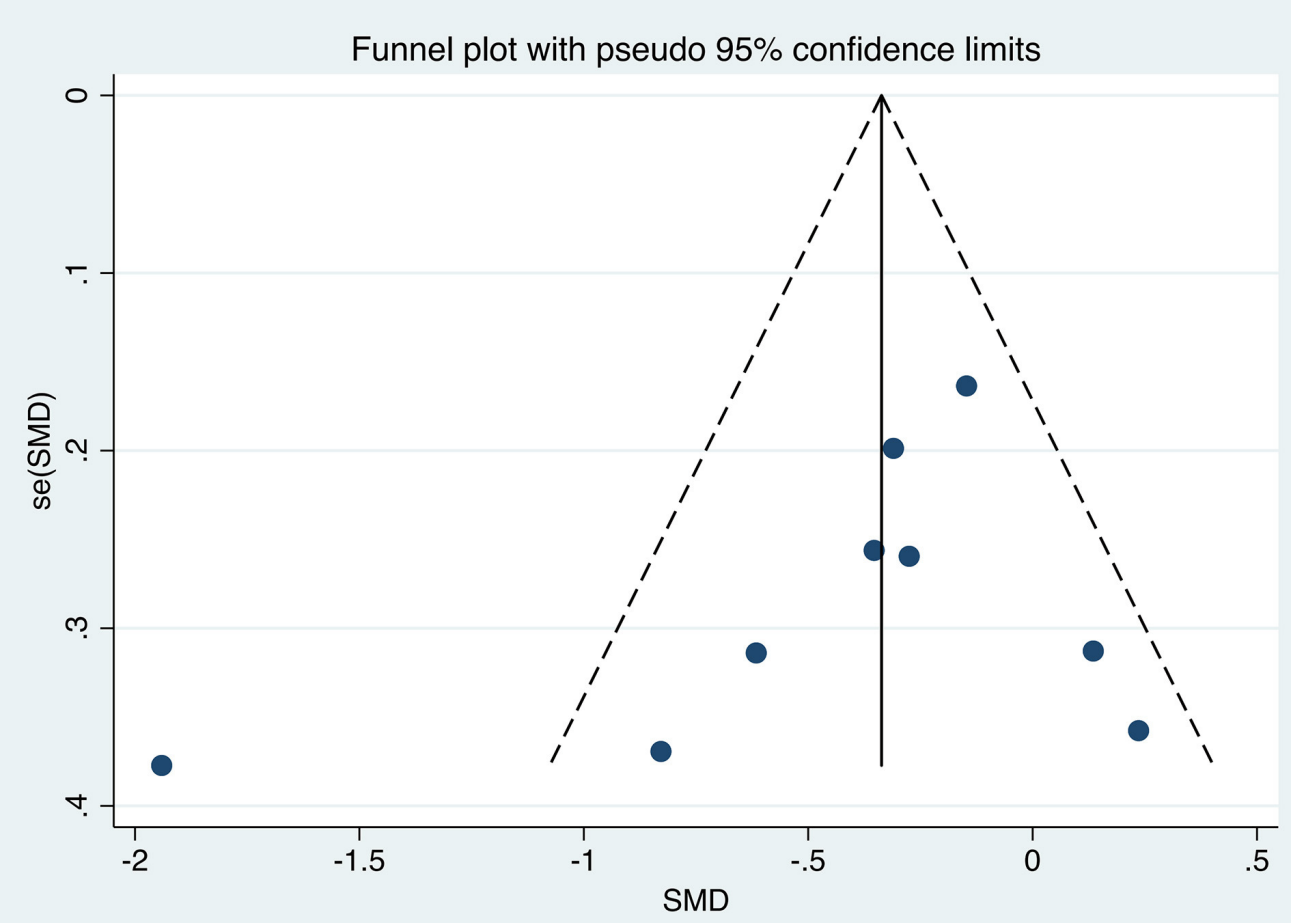
Funnel plot. SMD, Standard Mean Difference.

The efficacy of TMS intervention for reducing suicidal ideation was calculated for each study, as shown in Figure 2. The results of the meta-analysis showed that the suicidal ideation scores of the group who received TMS treatment for suicidal ideation were statistically significantly lower [standardized mean difference (SMD) = −0.415, 95% confidence interval (CI): −0.741 to −0.090, *P* = 0.012] than those of the control group. The heterogeneity of the studies was high [heterogeneity chi-squared (χ^2^) = 26.90, *P* = 0.001; *I*^2^ = 70.3%]. The funnel plot shown in Figure 3 reflects the publication bias by visual inspection. The results of Egger's test revealed no potential risk of publication bias (*t* = −1.25, *P* = 0.252).

#### Depression

**Figure 4:**
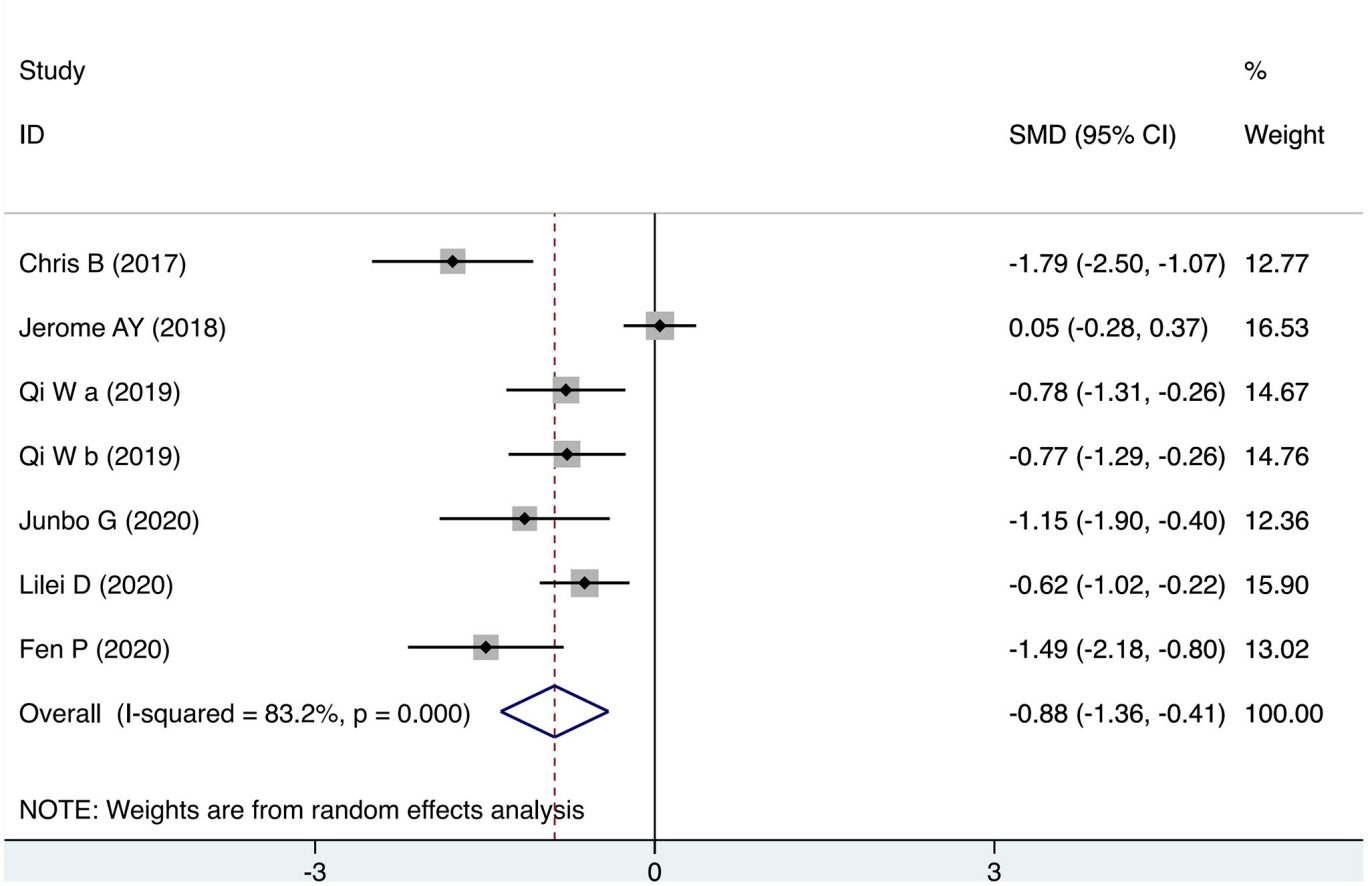
Forest plot of transcranial magnetic stimulation intervention for depression.

Seven studies provided scores for depression after the intervention. As shown in Figure 4, the meta-analysis results showed that the depression scores of the TMS group were statistically significantly lower than those of the control group (SMD = −0.885, 95% CI: −1.361 to −0.409, *P* = 0.012). The heterogeneity of the studies was high (heterogeneity χ^2^ = 35.67, *P* < 0.001; *I*^2^ = 83.2%).

### Subgroup Analysis

#### TMS Pattern

Meta-analysis using a random effects model showed that compared with iTBS (SMD = −0.207, 95% CI: −1.041 to 0.627, *P* = 0.627), the scores for suicidal ideation in patients who received rTMS intervention (SMD = −0.47, 95% CI: −0.849 to −0.091, *P* = 0.015) were significantly lower than those in the control group (Table 3).

**Table 3:** Subgroup analysis of included studies.

**Subgroup**		**No. of studies**	**Meta-analysis**		**Heterogeneity**	
			**SMD (95% CI)**	***P***	***I*^2^ (%)**	***P***
TMS pattern	rTMS	7	−0.47 (−0.849, −0.091)	*0.015*	74.50%	*0.001*
	iTBS	2	−0.207 (−1.041, 0.627)	0.627	68.80%	0.073
Age	<50	7	−0.498 (−0.972, −0.025)	*0.039*	75.70%	*<0.001*
	≥50	2	−0.213 (−0.460, 0.035)	0.092	0.00%	0.527
Intensity (% MT)	≤ 100%	5	−0.681 (−1.191, −0.171)	*0.009*	76.90%	*0.002*
	110%	2	−0.207 (−1.041, 0.627)	0.627	68.80%	0.073
	120%	2	−0.087 (−0.371, 0.198)	0.551	0.00%	0.424
Frequency	High	7	−0.382 (−0.782, 0.018)	0.061	76.00%	*<0.001*
	Low	2	−0.516 (−0.958, −0.074)	*0.022*	10.50%	0.291

*SMD, Standard Mean Difference. Italic values represent statistically significant results*.

#### Age

Meta-analysis using a random effects model showed that compared with age ≥50 years (SMD = −0.213, 95% CI −0.460 to 0.035, *P* = 0.092), suicidal ideation scores in the group aged <50 years who received the intervention (SMD = −0.498, 95% CI: −0.972 to −0.025, *P* = 0.039) were statistically significantly lower than those in the control group (Table 3).

#### Intensity Threshold

In three subgroups of intensity threshold 120%, 110%, and ≤ 100%, the efficacy of TMS intervention for suicidal ideation was represented by SMDs of −0.087 (95% CI: −0.371 to 0.198, *P* = 0.551), −0.207 (95% CI: −1.041 to 0.627, *P* = 0.627), and −0.681 (95% CI: −1.191 to −0.171, *P* = 0.009), respectively (Table 3).

#### Frequency

Meta-analysis using a random effects model showed that compared with the high frequency group (SMD = −0.382, 95% CI: −0.782 to 0.018, *P* = 0.061), the scores for suicidal ideation in the group who received a low frequency of rTMS (SMD = −0.516, 95% CI: −0.958 to −0.074, *P* = 0.022) were statistically significantly lower than those in the control group (Table 3).

### Cumulative Meta-Analysis

**Figure 5:**
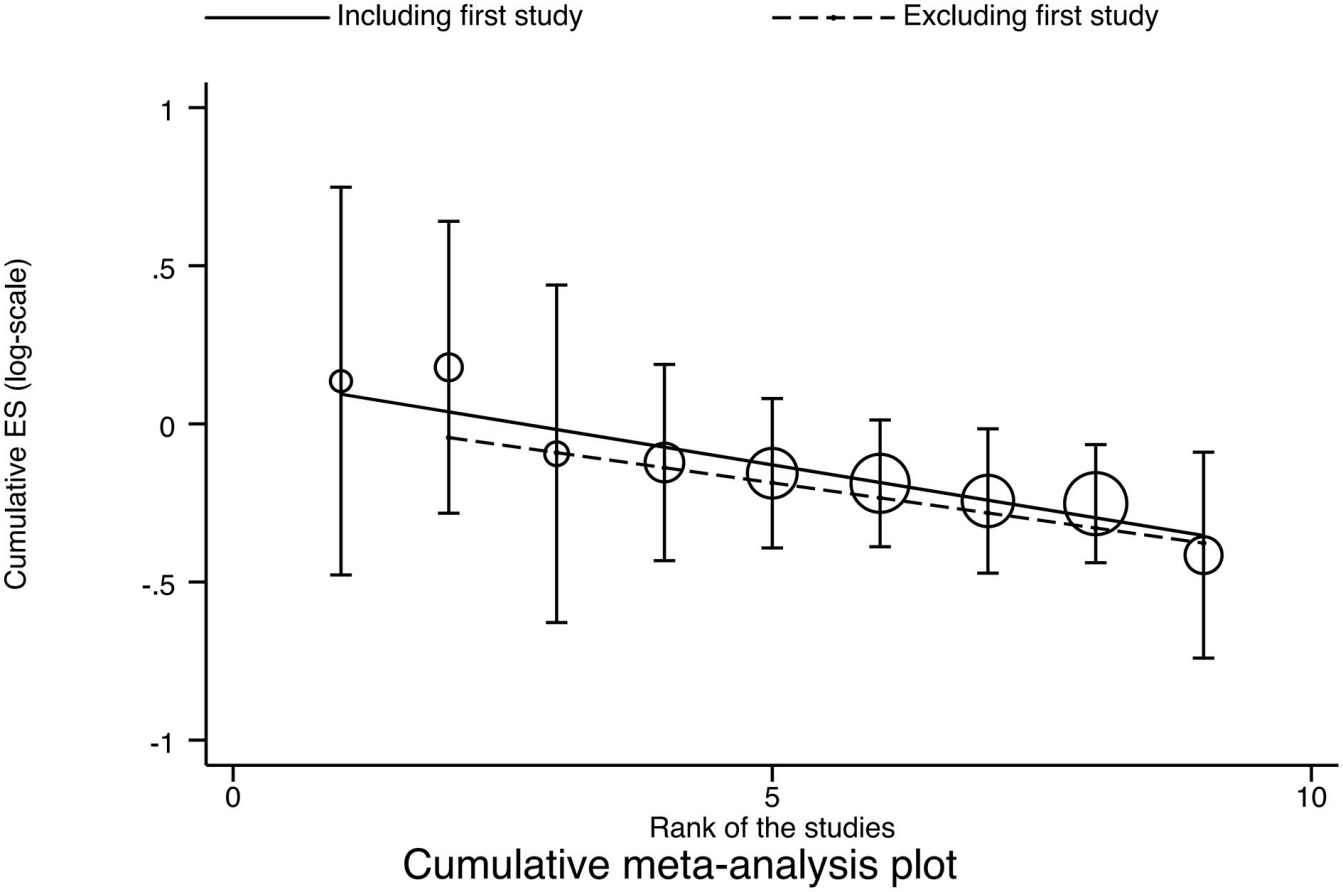
Cumulative meta-analysis plot. ES, Effect Size.

No obvious time trend was observed when applying the “initial vs. follow-up” strategy (*P* = 0.087) and regression strategy analysis (regression coefficient = −0.05585, *P* < 0.001). These results remained robust when the first study was removed and the results recalculated (regression coefficient = −0.04770, *P* < 0.001; Figure 5).

### Sensitivity Analysis

**Figure 6:**
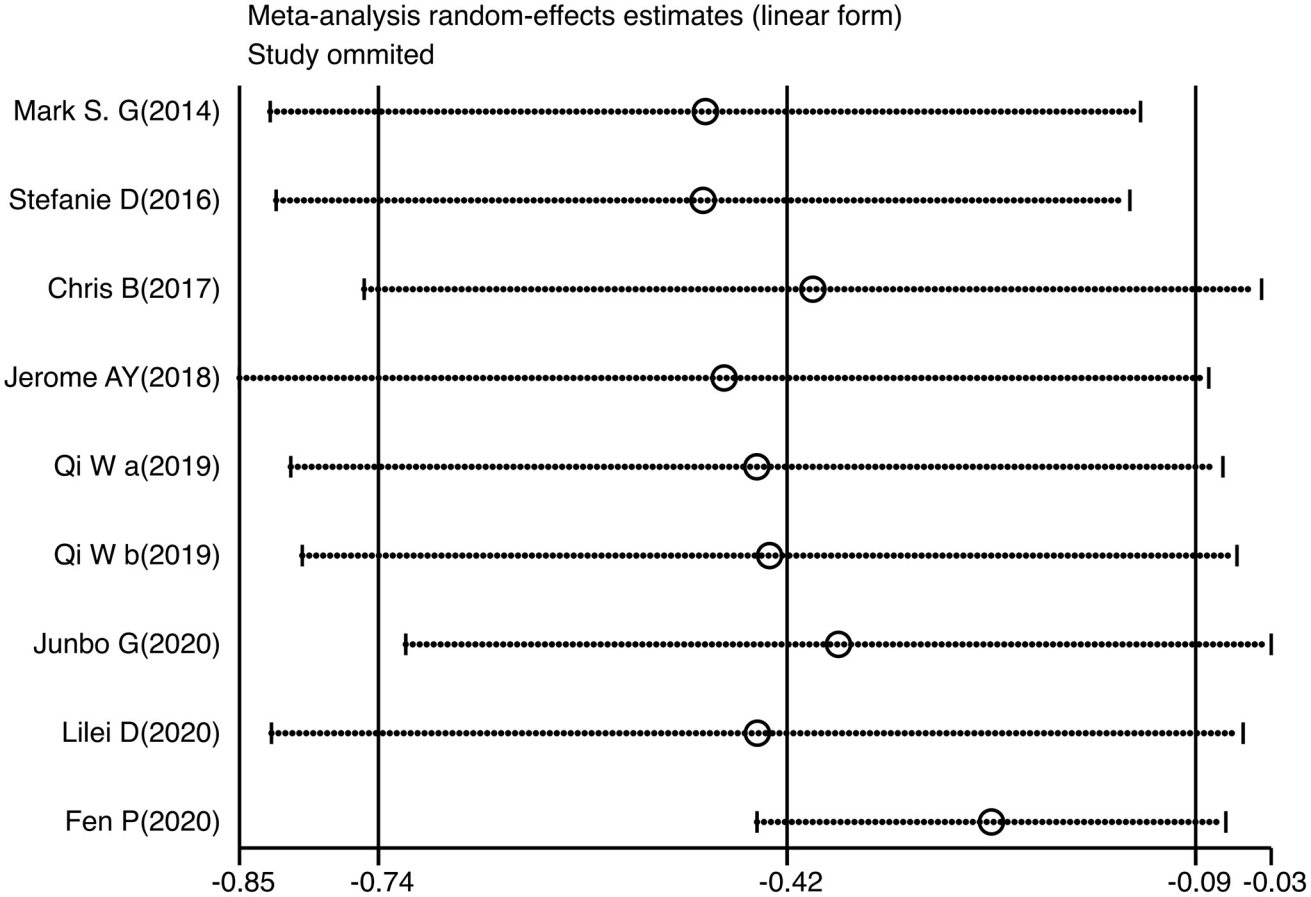
Influence analysis of individual studies.

Sensitivity analysis of the included studies showed that the point effect values fell within the 95% CI of the final effect values, which were stable and had no significant effect on the final conclusions (Figure 6).

## Discussion

The present study systematically reviewed the efficacy of TMS in reducing suicidal ideation in depression. The results showed that compared with the control group, the suicidal ideation scores of the group receiving TMS treatment were statistically significantly lower. The results showed that TMS was also significantly effective in alleviating depression. In summary, our survey of the existing research demonstrated that the use of TMS in managing suicide risk was promising, providing new evidence of the effectiveness and safety of TMS for alleviating suicidal ideation.

**Figure 7:**
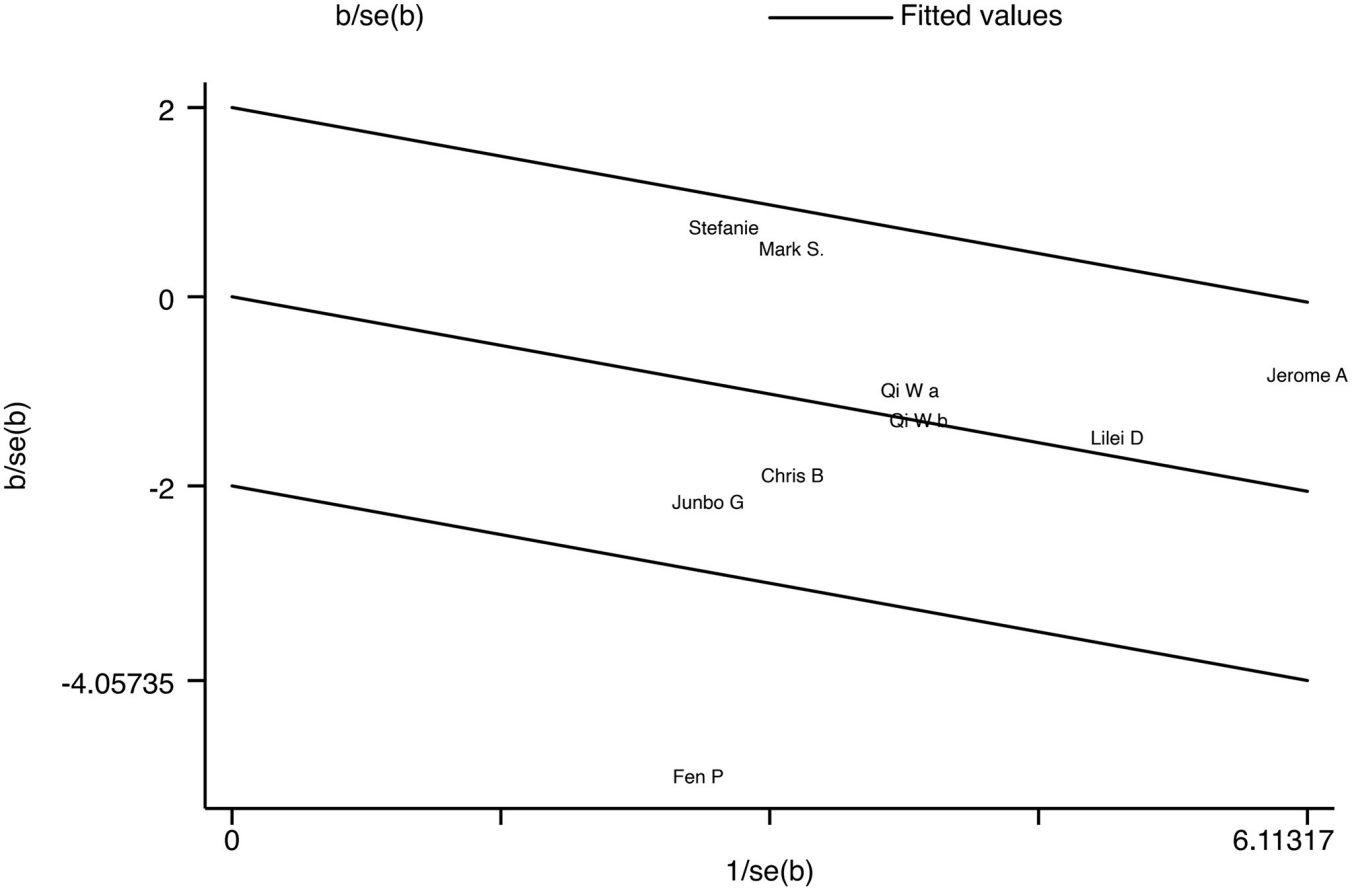
Galbraith star plot.

Our results showed moderate heterogeneity among the included studies. To explore possible influences on the effectiveness of TMS for reducing suicidal ideation, we performed subgroup analyses according to TMS pattern, age, threshold output rate, and frequency. The results show that these variables are indeed also a source of heterogeneity in this study. Heterogeneity was high (*I*^2^ = 70.3%), and the funnel plot showed that the outliers appeared to be from the same source (Figure 3). We found the article (38) that was the main source of heterogeneity and used it to draw a Galbraith star plot (Figure 7), and the SMD after excluding it was −0.252 (95% CI: −0.439 to −0.066, *P* = 0.008), with lower heterogeneity (*I*^2^ = 10.6%). A possible explanation for this might be that a novel neuro-navigation technique was used in that study to determine the coil location for TMS treatment, rather than using the traditional 5 cm method. This did not have an impact on the overall effect.

Although subgroup analyses were performed in our study, due to the limitations of the study size (non-significant groups are always small). Unfortunately, the results of subgroup analyses are likely to be unreliable in terms of bias. Of concern, this also suggests that future studies of TMS research interventions for suicide should pay more attention to the age of the subjects, different intervention modalities, and different parameter settings.

Cumulative analyses were performed in our study according to the time sequence of the studies, and meta-analysis repeated for each study added, reflecting dynamic trends in study results over time. The intervention effect was shown to be robust, and sensitivity analysis also indicated good stability (Sensitivity analyses were conducted by sequentially excluding studies with multiple daily sessions, and the results were that the apparent efficacy of TMS on suicidal ideation did not vary with the number of daily sessions and was robust).

TBS is a form of rTMS and these stimulation paradigms have been found to be safe in normal subjects and capable of producing consistent, rapid, and controllable electrophysiological, and behavioral changes (16, 39). However, no studies have shown that iTBS was a more effective intervention than rTMS in reducing suicidal ideation. Qin et al. who found that rTMS could achieve effective outcomes for older adults with depression, but that treatment outcomes were not as good as in young and middle-aged patients. In the study by wall et al., three adolescents had suicidal ideation and improved during TMS treatment (40). Stimulus intensity also influences the effectiveness of TMS interventions, as demonstrated by previous studies (41, 42). The rTMS may actuate, inhibit, or somehow otherwise interfere with the action of neuronal cortical networks, depending on stimulus frequency and intensity, and brain-induced electric field setup (43). But the relationship between the frequency and intensity of stimulation and the induced excitability change has not been extensively explored. In our study, the low frequency target was always on the right DLPFC, while the high frequency treatment target was always on the left DLPFC and low frequency treatment was more beneficial in reducing suicidal ideation scores in depressed patients than the higher frequencies. The effects of different stimulation frequencies on the cortex are not clear. It is commonly assumed that high frequency stimulation increases neuronal activity and cortical excitability in brain regions (44), and that low frequency stimulation decreases them (45). However, no consistent conclusions have been drawn about the therapeutic effects of different stimulation frequencies, and our conclusions on this were similar to those described by Chen et al. (46) and Lana et al. (47).

Compared to other physical interventions, such as ECT interventions, TMS interventions do not seem to be very effective (48). The most encouraging results supporting transcranial magnetic stimulation are those of studies (49–51). In a further study, DTMS was used in patients with severe TRD, via a new “H1” coil daily for 4 weeks. DTMS was associated with improvements in suicidal behavior (ideation and behavior), depression and related anxiety symptoms. The clinical safety of DTMS was also confirmed (52). Subjects in all included studies were depressed, and patients in two studies (33, 34) (iTBS) received physical interventions without medication. Combined with the results of the subgroup analysis of the TMS model, this seems to imply that the TMS intervention is promising as an adjunctive treatment. In one study, improvements were found in both suicidal ideation (especially in the first week of treatment) and depressive symptoms (50). An improvement in depressive symptoms was also shown in our meta-analysis of the results in depression (Figure 4). In a previous study, a reduction in suicide risk was found to be mediated by improvements in depressive symptoms (49). While in another study, changes in suicidal ideation were found to be unrelated to improvements in depression (33), Weissman et al. concluded that the correlation between depression and changes in suicidal ideation was 0.38 and suggested that suicidal ideation could be a specific target symptom construct for rTMS (53).

This study was designed to determine the effect of TMS treatment for reducing suicidal ideation in depression. Based on the above discussion, as well as the outcome indicators after our quantitative analysis, we believe that transcranial magnetic stimulation is promising in reducing suicidal ideation. The findings suggested that future research should focus more on low-intensity, low-frequency TMS interventions for suicidal ideation in middle-aged youth. However, due to the limitations of this study, this conclusion may warrant a serious warning, except to say that this study adds to the evidence for TMS interventions for suicidal ideation and provides a promising direction for future research on TMS interventions in large samples.

There were many limitations to this study. The unpublished literature was not searched, smaller sample subgroup analysis and the funnel plot suggested that publication bias may have resulted in an exaggeration of positive results. This study focused on suicidal ideation rather than suicidal behavior or attempts, and although SI is important, it is not the only factor that contributes to suicide. It is hoped that more future research will focus on the effects of TMS interventions on suicidal behavior or attempts. Finally, many studies have primarily included targeted treatment-resistant depression, and it is unclear whether SI in patients without TRD will show similar results. Despite its limitations, this is, to our knowledge, the first meta-analysis to quantitatively analyze the efficacy of transcranial magnetic stimulation on suicidal, which is arguably a strength of this study. And the study certainly added to our understanding of the efficacy of TMS intervention in reducing suicidal ideation in depression and provided valuable advice and direction for clinical treatment.

## Data Availability Statement

The original contributions presented in the study are included in the article/Supplementary Material, further inquiries can be directed to the corresponding author/s.

## Author Contributions

YC wrote the first draft of this manuscript and edited the subsequent versions. YC, HF, and XL are responsible for the data collection and analysis. CB, WG, XL, and FY gave critical revision for the manuscript. All authors contributed to the article and approved the submitted version.

## Funding

This work was supported by the National Natural Science Foundation of China (31771222) and the Natural Science Foundation of Anhui Province (KJ2016A355).

## Conflict of Interest

The authors declare that the research was conducted in the absence of any commercial or financial relationships that could be construed as a potential conflict of interest.

## Publisher's Note

All claims expressed in this article are solely those of the authors and do not necessarily represent those of their affiliated organizations, or those of the publisher, the editors and the reviewers. Any product that may be evaluated in this article, or claim that may be made by its manufacturer, is not guaranteed or endorsed by the publisher.
