# Supplementary material for: Efficacy of Transcranial Magnetic Stimulation for Reducing Suicidal Ideation in Depression: A Meta-Analysis
Source: Front Psychiatry. 2022 Jan 18;12:764183. doi: 10.3389/fpsyt.2021.764183 (PMC8803905; doi:10.3389/fpsyt.2021.764183)
Supplement: Supplementary file 1 [file Data_Sheet_1.docx]

**Supplementary materials**

PubMed search strategy:

(“Transcranial Magnetic Stimulation”[Mesh]) OR (Magnetic Stimulation, Transcranial[Title/Abstract]) OR (Magnetic Stimulations, Transcranial[Title/Abstract]) OR (Stimulation, Transcranial Magnetic[Title/Abstract]) OR (Stimulations, Transcranial Magnetic[Title/Abstract]) OR (Transcranial Magnetic Stimulations[Title/Abstract]) OR (Transcranial Magnetic Stimulation, Single Pulse[Title/Abstract]) OR (Transcranial Magnetic Stimulation, Paired Pulse[Title/Abstract]) OR (Transcranial Magnetic Stimulation, Repetitive[Title/Abstract]) AND ((“Suicide”[Mesh]) OR (Suicides[Title/Abstract]) AND (randomized controlled trial[Publication Type]) OR (randomized[Title/Abstract])) OR (placebo[Title/Abstract]).
